# Expression of concern: Schiff base Mn(iii) and Co(ii) complexes coated on Co nanoparticles: an efficient and recyclable magnetic nanocatalyst for H_2_O_2_ oxidation of sulfides to sulfoxides

**DOI:** 10.1039/d4ra90109f

**Published:** 2024-09-30

**Authors:** Shokoufeh Ghahri Saremi, Hassan Keypour, Mohammad Noroozi, Hojat Veisi

**Affiliations:** a Faculty of Chemistry, Bu-Ali Sina University Hamedan 65174 Iran; b Department of Chemistry, Payame Noor University Tehran Iran sho.saremi@gmail.com; c Center for Research and Development of Petroleum Technologies at Kermanshah, Research Institute of Petroleum Industry (RIPI) Iran

## Abstract

Expression of concern for ‘Schiff base Mn(iii) and Co(ii) complexes coated on Co nanoparticles: an efficient and recyclable magnetic nanocatalyst for H_2_O_2_ oxidation of sulfides to sulfoxides’ by Shokoufeh Ghahri Saremi *et al.*, *RSC Adv.*, 2018, **8**, 3889–3898, https://doi.org/10.1039/C7RA11225D.

*RSC Advances* is publishing this expression of concern in order to alert readers that concerns have been raised over the integrity of the data published in this article. The authors have been contacted but have not responded to requests to provide raw data. An expression of concern will continue to be associated with the article until a conclusive outcome is reached.

Signed: Laura Fisher

Date: 16th September 2024

Executive Editor, *RSC Advances*

